# Effects of inspiratory muscle training on respiratory function, diaphragmatic thickness, balance control, exercise capacity and quality of life in people after stroke: A randomized controlled trial protocol

**DOI:** 10.1371/journal.pone.0319899

**Published:** 2025-03-25

**Authors:** Fang Liu, Alice Y. M. Jones, Raymond C. C. Tsang, Timothy T. T. Yam, William W. N. Tsang

**Affiliations:** 1 Department of Physiotherapy, School of Nursing and Health Sciences, Hong Kong Metropolitan University, Hong Kong, China; 2 Department of Rehabilitation, Shenzhen Second People’s Hospital, The First Affiliated Hospital of Shenzhen University Health Science Centre, Shenzhen, China; 3 School of Health and Rehabilitation Sciences, The University of Queensland, Brisbane, Queensland, Australia; 4 Department of Rehabilitation Sciences, The Hong Kong Polytechnic University, Hong Kong, China; Università degli Studi di Milano: Universita degli Studi di Milano, ITALY

## Abstract

**Background:**

Stroke is associated with diaphragmatic weakness and impaired respiratory function as well as balance impairment. The role of inspiratory muscle training (IMT) on improvement of respiratory muscle strength in people after stroke has been reported. However, the training intensity associated with optimal diaphragm recruitment and the relationship between the effect of IMT and other functions in this population remains uncertain.

**Purpose:**

This randomized controlled trial (RCT) aims to explore the effects of a 4-week IMT program with training intensity at 50% maximum inspiratory pressure (MIP) (previously shown to be associated with maximal diaphragm contraction), on respiratory function, balance control, exercise capacity and quality of life (QOL) in people after stroke.

**Methods:**

This is a patient- and assessor-blinded RCT. Eligible participants will be randomly allocated to the targeted-IMT group (with 50% MIP as the training intensity) or sham-IMT group (with 10% MIP as the training intensity). Both groups will also receive the same standardised hospital-based physical-rehabilitation protocol. All interventions will be implemented 5 days/week for 4 weeks. The primary outcome is the change of diaphragmatic thickness. Secondary outcomes are spirometry respiratory function, balance control, exercise capacity and QOL. Assessment will be conducted at baseline, post-intervention, and at 12^th^ week follow-up. Data will be primarily analysed using repeated-measures ANOVA α=0.05.

**Discussion:**

Results of this study will primarily inform the effect of IMT on lung function, balance control, exercise capacity and QOL in addition to physical rehabilitation, further the interplay between the change in diaphragm thickness, balance and QOL will be analysed in detail.

## Introduction

Stroke is one of the most common causes of global morbidity and disability [1]. Besides the usual motor impairment, people after stroke may also encounter respiratory as well as diaphragmatic dysfunction [2]. The thickness of the diaphragm muscle on the stroke-affected side was reported to be only 50% of that in age- and gender-matched healthy adults [2]. The diaphragm muscle, while being the principal muscle for inspiration, also works synergistically with the pelvic floor, abdominal and back muscles contributing to trunk/core stability [3]. Therefore, weakness of the diaphragm, may be an important contributing factor to balance impairments in people after stroke.

Inspiratory muscle training (IMT) involves targeted exercises aimed at strengthening the inspiratory muscles, including the diaphragm, to enhance respiratory performance [4,5]. While the benefits of IMT have been explored in different populations [6–8], and reports on the effect of IMT in people after stroke on respiratory function were available [9,10], very few randomized controlled trials (RCTs) have specifically examined the impact of IMT on diaphragmatic function in this population [11,12], and the relationship between associated change of diaphragm thickness, balance outcome and quality of life (QOL) have not been thoroughly investigated. Further, published studies examining the relationship between IMT and diaphragmatic function, balance, and QOL in people after stroke have shown inconsistent results [12–15], likely due to varied training intensities of IMT programs [16]. The training intensity of IMT programs reported in the stroke population ranged from 30% to 80% maximum inspiratory pressure (MIP) [17,18]. Our recent studies have identified that an inspiratory load intensity at 50% MIP induced the highest diaphragmatic contraction both in healthy adults [16] and in the stroke population (under review). Simultaneous recording of diaphragm thickness (by ultrasonography) and sternocleidomastoid activity (by surface electromyography) in our previous work showed the diaphragm thickening fraction (DTf) was highest at an inspiratory load of 50%MIP, and the demand of inspiratory load above 50%MIP were primarily met by increased sternocleidomastoid activity but no or decreased diaphragmatic contraction [19]. This suggests that 50% MIP may be the optimal intensity for diaphragmatic training during an IMT program.

The aim of this current study is therefore to examine the additional effects of a 4-week IMT program, at intensity of 50%MIP, on the respiratory and diaphragmatic functions, balance control, exercise capacity, and QOL in people after stroke during their usual post-stroke rehabilitation management. Our hypothesis is that a 4-week target-IMT program will primarily improve diaphragm thickness and possibly associated with improvement in trunk balance, exercise capacity, and QOL.

## Methods

### Trial design

**Fig 1:**
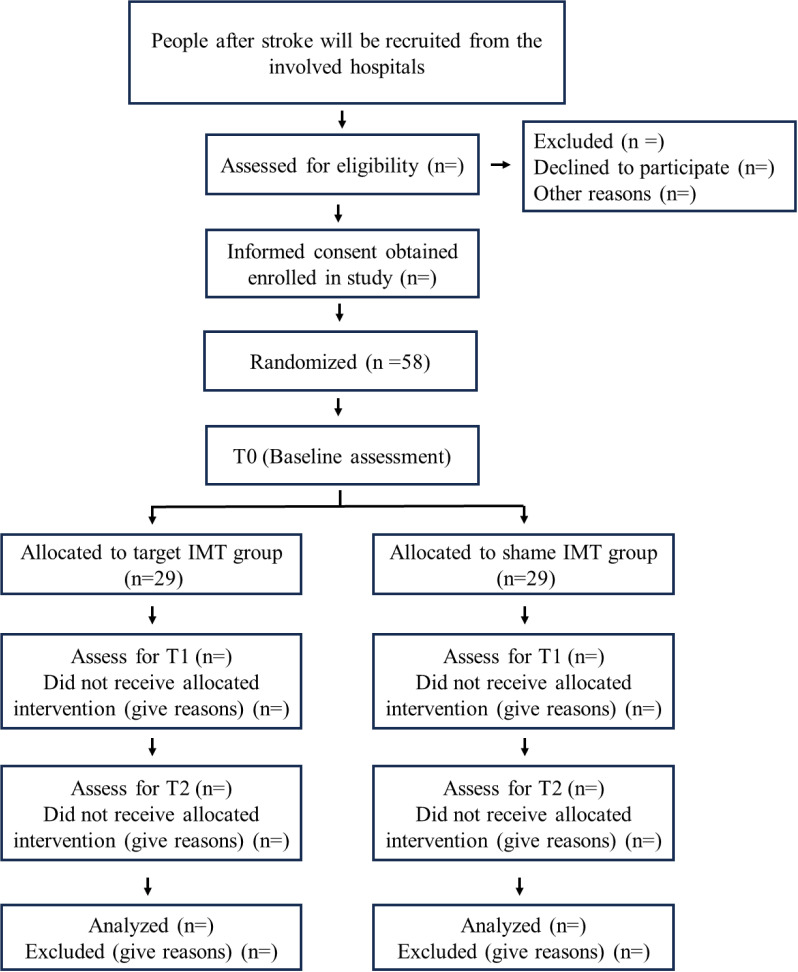
The flow diagram of participants.

This is a patient- and assessor-blinded, multi-center RCT. This study protocol is developed in compliance with the Standard Protocol Items of the Recommendations for Interventional Trials (SPIRIT) guidelines (S1 File) [20]. Patients will be randomly assigned to either the target-IMT or the sham-IMT group and receive respective training (see intervention below) for 4 weeks. Outcome measures (see below) will be recorded at three time points: at baseline before intervention (T0), after 4 weeks of intervention (T1), and at 12 weeks post-intervention (T2). The study procedure will follow the CONSORT flow diagram as shown in Fig 1.

### Ethics and dissemination

#### Ethics and trial registration.

Ethical approval was granted by the Research Ethics Committee of Hong Kong Metropolitan University (ethics approval number: HE-OT2023/13) and Shenzhen Second People’s Hospital (ethics approval number: 2023-274-01PJ). The study proposal was registered with ClinicalTrials.gov on 25th August 2024, with the registration number NCT06447272. Participants will be included after signing the informed consent form. Results will be submitted for publication in peer-reviewed journals.

#### Patient and/or the public involvement.

Patients and/or the public are not involved in the design, or conduct, or reporting, or dissemination plans of this research.

### Participants

People after stroke and meeting the inclusion criteria (Table 1) and receive rehabilitation treatment between 1 September 2024 to 31 December 2025 at several participating hospitals in Shenzhen, China, will be invited to participate in this study. Written informed consent will be obtained from all participants. The English example of the patient consent form and the study registration protocol is provided separately in S2 File and S3 File.

**Table 1:** The inclusion criteria and exclusion criteria.

Inclusion criteria
(1) Age ≥ 40 years and < 80 years; (2) breathing spontaneously; (3) clinically diagnosed with ischemic and/or haemorrhagic stroke; (4) duration of stroke from onset falls within 1 month to 12 months after diagnosis; (5) no thoracic or abdominal surgery within the last 6 months; (6) able to understand and follow verbal instructions; (7) no facial palsy, or mild facial palsy without limitation of labial occlusion; (8) able to maintain a resting sitting posture without feet support for at least 30 seconds; (9) no cognitive impairment (Montreal Cognitive Assessment (MoCA) score ≥ 26); (10) able to independently walk at least 10 meters with or without an assistive device.
Exclusion criteria
(1) acute myocardial infarction or acute heart failure; (2) acute pain in any part of the body; (3) with respiratory illness or positive clinical signs of impaired respiratory function (such as shortness of breath, hypoxemia, chronic cough and sputum retention); (4) with chronic cardiovascular dysfunction; (5) Trunk Impairment Scale (TIS) score ≥ 20. (6) patient with a nasal feeding tube, tracheal tube and/or any condition that prevents the measurement or the implementation of the study procedure.

### Sample size calculation

Sample size calculation will be based on the use of independent t-test using the G * Power 3.1. 9.7. The calculation was based on the hypothesis that a statistically significant difference in the change of maximum diaphragmatic thickness on the affected side (primary outcome) would be observed between the intervention group and the control group.

Based on the similar study conducted by Cho et al [12], the effect size for the between-group difference in diaphragmatic thickness during maximum contraction of the affected side diaphragm is 0.843. Using a two-sided alpha level of 0.05 and a power of 0.80, the estimated sample size is 24 participants per group. To account for a potential 20% attrition rate, an additional 20% was added, resulting in a final required sample size of at least 29 participants per group (total n =  58).

### Randomization, allocation concealment and blinding

Patients will be randomized into either the target-IMT group or the sham-IMT group. The random sequence is generated using the randomly permuted blocks with a size of 4 per block. Blocks are specified as AABB, ABAB, ABBA, BBAA, BABA, BAAB (A = sham-IMT group, B = target-IMT group). To eliminate the distribution discrepancies of gender (male and female), duration (subacute and chronic stroke), and age (above or below 65 years) between both groups at baseline, stratified blocked randomization will be performed to balance the number of these three prognostic factors [21]. Eight strata will be generated. An independent researcher who is not involved in the participant recruitment, delivery of intervention and outcome assessment will randomly select the blocks for each stratum and generate the final allocation sequence. The random allocation will be maintained in different sealed opaque envelopes and stored in a locked cabinet by the same researcher. When an eligible participant is recruited into the trial, the therapist will contact the independent researcher to open the sealed envelope to determine the allocation of the participant.

Participants will be blinded to the study group allocation but informed that they will receive an IMT program with different inspiratory loads. The display interface of the IMT training devices will be covered with opaque material, so that the participant will be blinded to the intensity of training. To prevent allocation leakage and minimize the possibility of participants interacting between patients, all IMT sessions will be conducted in separate rooms.

The assessors assigned to collect data will be blinded to the group allocation of the participants. In case of serious adverse events (SAEs) or suspected unexpected serious adverse reactions (see below), the participant blinding status will be revealed after consultation with the principal investigator (PI). Subsequently, these events will be reported to the Ethical Committees of the primary hospital and the university involved.

### Interventions

Patients will be randomized into Group A (sham-IMT) and Group B (Target-IMT). Both groups will receive conventional treatment and threshold IMT, but with different IMT intensities. Group A will receive sham-IMT, with a training intensity set at 10% of MIP [22]. Group B will receive targeted IMT (with 50% MIP as the training intensity, an intensity that was shown in our previous study to be associated with highest diaphragmatic contraction). Adverse events (see below) will also be recorded during the intervention.

#### The conventional rehabilitation treatment.

Participants in both groups will receive a standardised conventional rehabilitation protocol. It includes limb range of motion, muscle tone reduction, strengthening and endurance of limb muscles, transfer skills, task-directed movements, general gait training, and activities of daily living training [23]. This standardised protocol will be implemented for 60 minutes per session, 5 days per week, for 4 weeks.

#### IMT treatment.

A threshold inspiratory loading device (POWERbreathe, KH2, UK) will be used during the IMT program to ensure that all participants receive the ‘set’ resistance. The training will be supervised by physiotherapists, who has more than 3 years of clinical experience in respiratory management of people after stroke. All physiotherapists participating in delivering the intervention will be trained prior to the trial to ensure consistency.

All participants will receive 3 IMT sessions per day (morning, afternoon and evening). Each session will consist of 5 sets of 10 breaths per set, at 50%MIP, with one-minute rest intervals between sets. The total duration will be around 15 minutes per session. Training will be conducted 5 days per week for 4 weeks. Therefore, each patient will receive a total of 60 IMT sessions during the whole 4-week intervention.

For Group A (sham-IMT group), the intensity of IMT will be kept at 10% of MIP, and no readjustment of the inspiratory load throughout the 4 weeks. For Group B (target-IMT group), the training intensity will be maintained at the 50% MIP with weekly reassessment of MIP. Adjustment of training load will be made according to any change in the MIP from the weekly reassessment.

### Outcome measures

Data collection will be performed by another physiotherapist, who is blinded to the group assignment and not involved in the delivery of interventions to any participants. Demographic data of each participant, including age, gender, height, weight, and body mass index (BMI), will be recorded for all participants. Additionally, information regarding stroke type (cerebral infarction, intracerebral haemorrhage), duration from the first onset of stroke, and medical history (hypertension, diabetes mellitus, lipidemia, kidney disease), the National Institutes of Health Stroke Scale (NIHSS), Modified Rivermead Mobility Index (MRMI), Modified Rankin Scale (mRS), and Barthel Index (BI) will all be retrieved from the patient’s medical records.

The primary outcome is the change of diaphragm thickness at different measurement time points, and the secondary outcomes are a) respiratory function [measured by spirometry including MIP, forced vital capacity (FVC) and forced expiratory volume in one second (FEV1)]; b) balance control – reflected by multiple assessment tools including a force plate to measure changes in the center of pressure (COP) during various tasks performed in a sitting position; the Trunk Impairment Scale (TIS); the Timed Up-and-Go test (TUG); and the Falls Efficacy Scale–International (FES-I); c)Exercise capacity will be reflected by the distance covered in the 6-minute walk test (6MWT); d) QOL measured with Stroke Impact Scale 3.0 (SIS)).

In view of the diversity in assessment tools reported in evaluation of balance in people after stroke, multiple balance assessment tools are considered in this study to allow a wider scope of comparison of our results with that published in the literature. The use of a force plate will be employed in evaluation of COP during dynamic sitting tasks, this provides more credible data than distance associated with postural sway.

#### Diaphragmatic thickness.

Ultrasonographic scans (Mindray M9, Shenzhen, China) will be used to measure the diaphragmatic thickness. The ultrasonography measurement will be conducted by an assessor who had received proper training from a radiologist specialized in ultrasonography.

Participants will be requested to maintain an upright sitting position on a chair with their back supported throughout this measurement. The transducer probe with a bandwidth of 4-12Hz in B mode will be used for diaphragmatic thickness measurements. Diaphragm thickness on both sides will be measured, in consequence order, by the same assessor with the probe to be placed on the intercostal space of the eighth and ninth intercostal spaces, between the anterior-axillary and mid-axillary lines, and with a 45° angle tilt towards the surface of the abdominal wall in the cephalic direction. The diaphragmatic thickness at the end of tidal inspiration, the end of tidal expiration and the end of maximum expiration will be recorded. Three images of each breathing status will be captured and averaged to determine the final diaphragmatic thickness.

#### Center of Pressure (COP).

A FreeMed force platform (Sensor Medica, Guidonia Montecelio, Roma, Italy) will be used to measure the balance control capacity of all participants. The sampling frequency will be set at 25 Hz in real time following instructions provided by the manufacturer. Assessment of sitting balance will be conducted with a force plate placed on a rigid wooden table, and the participant will be required to sit on the force plate with arms crossed over their chest. No support will be provided over the participant’s back or feet. After making the initial positioning adjustments, participants will be asked to sit quietly for 30 seconds to measure static sitting stability. After that, the participant will be asked to perform 4 different tasks with eyes open including forward trunk flexion, backward trunk extension, left lateral trunk flexion, and right lateral trunk flexion. The sequence of these tasks will be randomized. During each task, participants will be instructed to move the trunk as far as possible in each direction without compromising their balance. Participants are permitted to adjust the speed of trunk movement according to their comfort level. All tasks will be repeated three times. The sway area during quiet sitting and the sway path of each trunk reach test of the COP will be obtained using the software Free Step v.1.6.5 and the data recorded with the best performance in each task will be adopted for final data analysis.

#### Respiratory function.

Spirometry including FVC and FEV_1_ will be performed using a spirometer (XEEK, X1, China). MIP will be evaluated using another spirometer (POWER breathe, KH2, UK). All lung function tests will be conducted in accordance with the guideline recommendations by the American Thoracic Society [24]. Each test for each outcome measurement will be required to be performed at least three times, with no variation exceeding 5% or 100 millilitres between tests. The highest value from the three trials will be adopted for the final analysis [25].

#### Trunk Impairment Score (TIS).

TIS is a clinical assessment tool used to evaluate and quantify trunk control and stability in individuals with neurological conditions, particularly those who have suffered a stroke [26]. This scale consists of 3 subscales of static and dynamic sitting balance and trunk coordination [26]. The static sitting balance subscale assesses whether a person can sit independently and remain seated when the legs are either passively or actively crossed. The dynamic sitting balance subscale evaluates the ability to actively shorten each side of the trunk. Trunk coordination tests the ability to independently rotate the shoulder girdle and pelvic girdle. The total TIS score ranges between 0 and 23 points, with a higher score indicating better trunk function. The reliability of TIS for individuals after stroke has been reported in previous study [27].

#### Timed- Up-and Go test (TUG).

TUG is a test to measure the time that a participant needs to stand up from a standard armchair, walk a short distance of about 3 meters, turn around, walk back to the chair, and sit down again. This test is widely used to assess mobility and risk of falls in older adults and individuals with neurological conditions. The reliability and validity of this test in people after stroke have been supported [28].

#### Falls Efficacy Scale–International scale (FES-I).

The Chinese version of the FES-I will be used in this study to measure the person’s level of concern (confidence) about potential falls during activities within the home and community [29]. The FES-I has been shown to have good reliability and validity in measurement [30]. The FES-I contains 16 items. The total scores range from 16 to 64 points, with higher scores indicating lower self-efficacy [30].

#### 6-Minute Walk Test (6MWT).

The exercise tolerance of patients will be assessed by the 6-minute walk test (6MWT). The 6MWT will be performed in a 30-meter hospital hallway. The 6MWT is conducted according to the standard protocol recommended by the American Thoracic Society (ATS) [31]. In order to accommodate the learning effect, this test will be performed twice with at least a 30-minute interval, and the larger distance recorded will be used for the final statistical analysis.

#### Stroke Impact Scale 3.0 (SIS 3.0).

The Chinese version of the Stroke Impact Scale 3.0 (SIS 3.0) will be used to measure the QOL. The validity and reliability study of the Chinese version of SIS 3.0 has been well documented [32]. The SIS 3.0 is a 59-item questionnaire, with items under subheadings in eight domains: strength (4 items); memory and thinking (7 items); emotion (9 items); communication (7 items); specific functional tasks (10 items); mobility (9 items); hand function (5 items); and participation and role function (8 items). Each item is rated on a five-point Likert scale in terms of the difficulty the patient has experienced in completing each item. In addition, an extra question related to functional stroke recovery asks the patients to rate, on a scale from 0 to 100, how much he/she has recovered from a stroke, with zero being the worst result and 100 being the best.

All outcomes will be measured at the baseline (T0) before randomization and after 4 weeks of intervention (T1). To obtain more information on prolonged effect of the program on functional improvement, TIS, TUG, FES-I, and SIS will be measured at 12 weeks after intervention (T2). It is anticipated that majority of the patients reside in a different city after discharge, in order to reduce the dropout rate for post-discharge follow-up, all follow-up data will be collected using tele-assessment through WeChat, an online chatting application in China. Prior to discharge, the patients will be instructed how to conduct the measurement at home.

#### Adverse events/SAEs assessment and management.

Safety will be reported as adverse events (AEs) or SAEs. The assessment, recording, and management of AEs/SAEs are illustrated in S4 File.

#### Data management and safety monitoring.

All baseline data and raw data will be promptly recorded on CRFs in a complete, accurate, and clear manner upon data acquisition. Two assessors will cross-check to ensure the accuracy of the data. A Microsoft Excel spreadsheet will serve as the database for managing the data. All study data will be promptly entered into this electronic database. Data input and proofreading will be conducted by two independent researchers, leading to double data entry and storage.

Upon enrolment, every participant will receive a distinct numeric study ID for anonymous tracking throughout the study. Access to identifiers linking data to individual participants will be restricted solely to the PI. Consent forms and hard copy data collection forms will be securely stored in a locked cabinet at the research center, with access granted only to the PI.

### Statistical analyses

All data will be analyzed using the IBM SPSS Statistics for Windows, Version 25.0 (Armonk, NY: IBM Corp). Intention-to-treat principle will be used to analyze the data. Missing data of each time point will be imputed using multiple imputation method [33]. Repeated-measures ANOVA will be performed to compare the changes of diaphragmatic thickness, respiratory function, balance control, exercise capacity, and QOL, respectively. If the interaction effect of repeated-measures ANOVA is significant, post-hoc multiple comparisons will be conducted with Bonferroni correction to assess the interaction effect between groups and different time points. If the final data for any outcome do not meet the Gaussian assumption, non-parametric tests (e.g., Friedman’s test) will be used to compare the differences. A significance level of 0.05 will be applied for all analyses.

#### Dissemination plan.

The results of this study are expected to be submitted in international peer-reviewed journals in 2025.

## Discussion

This RCT is designed as a patient-blinded and assessor-blinded study to explore the effect of a 4-week IMT program with training intensity set at 50%MIP, in addition to a traditional physical rehabilitation program on diaphragmatic and respiratory functions, and its potential in enhancing balance, exercise capacity, and QOL. The selected training intensity was based on a our previous findings from both on healthy adults [16] and in a cohort of people with stroke. We have demonstrated that highest diaphragmatic contraction was associated with an inspiratory load resistance at 50%MIP, both in healthy adults [16] and in a cohort of people after stroke (manuscript under review).

In previous studies, IMT was found to significantly improve balance control, reflected by improved TIS rather than BBS [13–15]. Although TIS has been shown to be more sensitive and specific to assess trunk balance than BBS [34], TIS has been criticised for that it is a subjective scale susceptible to influence by the rater. The effect of IMT on balance as reflected by changes in sway area and shifts of COP in people after stroke has not been investigated. This study aims to provide information on the effect of IMT on static and dynamic sitting balance tasks, reflected by objective data recorded by a force plate.

People after stroke who present with inspiratory muscle weakness were found to have lower walking speed [35], but the findings of improvement in walking ability after IMT were controversial. In Back and Kim’s study, IMT was found to be associated with improved 6MWD [36], while no significant changes in 6MWD were observed in other studies [12,37,38]. One possible reason for this discrepancy is that the improved diaphragm strength after IMT might not be adequate to yield a notable enhancement in exercise capacity. On the other hand, only two studies reported the change of diaphragmatic function after IMT intervention [12,39]. In Cho’s study [12], although IMT did not demonstrate an effect on 6MWD, DTf of the affected side improved by 38% compared to the control group without IMT [12]. In accord with Cho’s study, Jung et al [39] also demonstrated a significant improvement in DTf by 55% on the affected side. However, in both Cho and Jung’s study [12,39], 30% MIP was used as the training intensity for 6 weeks and with only one session per day. In this current RCT proposal, a higher intensity of 50% MIP will be adopted, while the duration of the program will only be 4 weeks, each participant is expected to be trained 3 sessions per day. By comparing with previous studies, results of this proposed study will provide further information on training effects associated with a higher intensity and training frequency, but more importantly, the interplay between diaphragm function, balance control and exercise capacity will be explored in more detail.

The proposed study is innovative in several ways:

1. **Focus on Diaphragmatic Function**: Unlike previous studies that primarily focused on respiratory outcomes, this study specifically targets the change in diaphragmatic thickness, which potentially affect both respiratory function as well as trunk stability.
2. **Standardized IMT Intensity**: The IMT intensity adopted in this RCT was determined from findings of two pilot studies, providing evidential support of direct relationship between inspiratory training load and diaphragmatic contraction.
3. **Comprehensive Outcomes**: The study aims to assess a wide range of outcomes, including respiratory and diaphragmatic functions, balance control, exercise capacity, and QOL. This comprehensive approach provides a holistic view of the potential benefits of IMT.
4. **Potential for Broader Application**: Although the study focuses on stroke survivors, its findings could potentially be applied to other populations with similar respiratory and diaphragmatic impairments, such as individuals with chronic obstructive pulmonary disease (COPD) or spinal cord injuries.

Although this study is a multi-center RCT, the whole trial will be conducted only in Shenzhen, China, this may limit the generalizability of its findings. Future research that includes more geographical locations and larger sample sizes will still be necessary.

## Supporting information

S1 File The SPIRIT-Checklist. (DOCX)

S2 File Consent form. (DOCX)

S3 File The registration study protocol. (DOCX)

S4 File Adverse events or serious adverse events assessment and management. (DOCX)

## Abbreviations

AEs: Adverse events
ANOVA: Analysis of VARIANCE
BI: Barthel Index
BMI: Body mass index
COP: Center of pressure
DTf: Diaphragm thickening fraction
FES-I: Falls Efficacy Scale–International scale
FEV_1_: Forced expiratory volume in one second
FVC: forced vital capacity
IMT: Inspiratory muscle training
MIP: Maximum inspiratory pressure
mRS: Modified Rankin Scale
MRMI: Modified Rivermead Mobility Index
NIHSS: National Institutes of Health Stroke Scale
PI: Principal investigator
QOL: Quality of life
RCT: Randomized controlled trial
SAEs: Serious adverse events
SPIRIT: Standard Protocol Items of the Recommendations for Interventional Trials
SIS: Stroke Impact Scale 3.0
TUG: Timed up-and-go test
TIS: Trunk Impairment Scale
6MWT: 6-minute walk test
6MWD: distance of 6-minute walk test
