# Supplementary material for: Effects of inspiratory muscle training on respiratory function, diaphragmatic thickness, balance control, exercise capacity and quality of life in people after stroke: A randomized controlled trial protocol
Source: PLoS One. 2025 Mar 25;20(3):e0319899. doi: 10.1371/journal.pone.0319899 (PMC11936291; doi:10.1371/journal.pone.0319899)
Supplement: S2 File — (DOCX) [file pone.0319899.s002.docx]

**S2 File. Consent form**

**HONG KONG METROPOLITAN UNIVERSITY**

**Information Sheet**

**TITLE OF THE STUDY**

Effects of inspiratory muscle training on respiratory function, diaphragmatic thickness, balance control, exercise capacity and quality of life in people after stroke: a randomized controlled trial

**INTRODUCTORY SENTENCE**

You are invited to participate in a research study conducted by Prof. William Tsang Wai Nam from the Department of Physiotherapy, School of Nursing and Health Studies of Hong Kong Metropolitan University (HKMU).

**PURPOSE OF THE STUDY**

To investigate the effects of inspiratory muscle training on respiratory function, diaphragmatic function, balance control, exercise capacity and quality of life in people after stroke.

**PROCEDURES**

If you are willing to participate in this study, you will be randomized into either Group A or Group B. Both groups will receive the same conventional treatment and additional inspiratory muscle training, but the intensity of the inspiratory muscle training will differ. Each group will undergo 4 weeks of their respective training (5 times/week, 1.5 hours/day). To evaluate your functional changes, we will measure your lung function, diaphragmatic function, balance control, walking capacity, and quality of life before and after the 4-week intervention. Some measurements will be measured again through an online application (WeChat) at the 12th week after the intervention. All measurements are non-invasive, and each assessment will last around 1.5 hours.

**POTENTIAL RISKS/STRESS/PAIN/DISCOMFORTS/OTHER FACTORS AND THEIR MINIMIZATION**

There will be no direct risk, stress, pain or discomforts in participating in this study.

**POTENTIAL BENEFITS**

All measurements described above will be free of charge. Through our assessments, you will gain a comprehensive understanding of your health status. Additionally, we will provide you with a threshold inspiratory training device at no cost. After you complete the whole experiment, if you wish to continue using this device for training, we will offer you free guidance.

**PARTICIPATION AND WITHDRAWAL**

You have every right to withdrawn from the study before or during the measurement without penalty of any kind.

**CONFIDENTIALITY**

Your personal information and data will not be disclosed to any person not being in the research team. Your name or photo will not appear on any published materials.

**QUESTIONS AND CONCERNS**

If you have any questions or concerns about the research study, please feel free to contact Prof William Tsang Wai Nam of HKMU at 3970 8703. If you have questions about your rights as a participant of this research study, please contact the Research Ethics Committee of HKMU at 2768 6251.

**Consent Form**

Hong Kong Metropolitan University

School of Nursing and Health Studies, Department of Physiotherapy

Consent form for

**Effects of inspiratory muscle training on respiratory function, diaphragmatic thickness, balance control, exercise capacity and quality of life in people after stroke: a randomized controlled trial**

I have read and understand the information provided about the above study. I agree to participate this study.

|  |  |  |
| --- | --- | --- |
| Name of participant | Signature of participant | Date |

|  |  |  |
| --- | --- | --- |
| Name of investigator | Signature of investigator | Date |
