# Supplementary material for: Effects of inspiratory muscle training on respiratory function, diaphragmatic thickness, balance control, exercise capacity and quality of life in people after stroke: A randomized controlled trial protocol
Source: PLoS One. 2025 Mar 25;20(3):e0319899. doi: 10.1371/journal.pone.0319899 (PMC11936291; doi:10.1371/journal.pone.0319899)
Supplement: S3 File — (DOCX) [file pone.0319899.s003.docx]

**S3 File. The registration study protocol**

**Effects of inspiratory muscle training on respiratory function, diaphragmatic thickness, balance control, exercise capacity and quality of life in people after stroke: a randomized controlled trial protocol**

**Background of the study**

Most people after a stroke present with impaired balance function, which is associated with poor recovery of activities of daily living, mobility, and an increased risk of falls. Hence, improving the balance function is one of the main goals of stroke treatment.

Respiratory muscular weakness especially the diaphragm weakness has also been found to be associated with a decreased balance function. Diaphragm not only as the principal muscle for inspiratory, but also plays an important role in balance control. The contraction of diaphragm increases intra-abdominal pressure, working synergistically with the pelvic floor and abdominal muscles to increase trunk stability.

Inspiratory muscle training is an intervention that has been used in people after stroke to train the strength and endurance of inspiratory muscles. However, the present intensities commonly range between 30% of maximum inspiratory pressure (MIP) to 80% MIP. However, in the studies of healthy adults, it has been reported that a higher intensity does not necessarily associate with more forceful diaphragm muscle contractions. Hence, optimum intensity especially for training diaphragm function for people after stroke is unknown.

Our recent studies have identified that an inspiratory load intensity at 50% MIP induced the highest diaphragmatic contraction both in healthy adults and in the stroke population. This RCT study will be based on this optimal IMT intensity (50% MIP) to explore: the effects of a 4-week protocol of inspiratory muscle training (IMT) on respiratory function, balance control, exercise capacity, and quality of life in people after stroke.

**Methods**

**Ethical approval**

Ethical approval has been received from the Research Ethics Committee of Hong Kong Metropolitan University (ethics approval number: HE-OT2023/13) and Shenzhen Second People’s Hospital (ethics approval number: 2023-274-01PJ).

**Sample size calculation**

Sample size calculation will be based on the use of analysis of variance (ANOVA) using the G*Power 3.1. Considering an effect size of 0.843, an alpha error probability of 0.05 and a power of 0.80, the estimated sample size is 24 for each group. An addition of 20% of the sample size will be included to adjust for possible attrition. The final sample size required will be at least 29 for each group (total n= 58).

**Participants**

Inclusion criteria for people after stroke:

(1) aged between 40 and 80 years; (2) breathing spontaneously; (3) clinically diagnosed with ischemic and/or haemorrhagic stroke; (4) stroke duration from onset between 1 and 12 months; (5) no history of thoracic or abdominal surgery within the last 6 months; (6) able to understand and follow verbal instructions; (7) no facial palsy, or mild facial palsy without limitation of labial occlusion; (8) capable of maintaining a resting sitting posture without feet support for at least 30 seconds; (9) no cognitive impairment, as indicated by a Montreal Cognitive Assessment (MoCA) score of ≥ 26; and (10) able to independently walk at least 10 meters with or without an assistive device.

Exclusion criteria for people after stroke:

(1) acute myocardial infarction or acute heart failure; (2) acute pain in any part of the body; (3) with respiratory illness or positive clinical signs of impaired respiratory function, such as shortness of breath, hypoxemia, chronic cough, or sputum retention; (4) with chronic cardiovascular dysfunction; (5) Trunk Impairment Scale (TIS) score ≥ 20; or (6) presence of a nasal feeding tube, tracheal tube, or any condition that prevents the measurement or implementation of the study procedure.

**Procedure**

**Treatment**

Patients will be randomized into two groups (group A and group B). Group A will receive conventional treatment + sham IMT (with 10% maximum inspiratory pressure （MIP） as the intensity) and Group B will receive conventional treatment + target IMT (The intensity of IMT will be determined based on the results of our undergoing study, which was registered on ClinicalTrials.gov with the registration number NCT06267768)

The conventional treatment

The conventional treatment includes limb muscle strengthening exercises, therapy using the Bobath approach, general gait training and occupational therapy for 60 minutes/day, 5 days/week, for 4 weeks.

IMT treatment

All participants will be trained thrice daily for three sessions (morning, afternoon and evening, separately) of IMT. Each session will consist of 10 breaths per set for a total of 5 sets with one-minute rest intervals between sets. Total duration to be around 15 minutes per session.

Training will be conducted 5 days per week for 4 weeks.

After the 4 weeks IMT program, 12 weeks of follow-up will be arranged.

**Blind**

Random allocation will be performed with block randomization and maintained in different sealed opaque envelopes by a physiotherapist who is not involved in this study. Participants will be blinded to the study group allocation but informed that they will receive an IMT with a different inspiratory load. The display interface of the IMT training devices will be covered with opaque material, so that the participant and the physiotherapist who conducts the IMT training will be blinded. The assessors will also be blinded to the allocation of the participants.

**Measurements**

**Lung function**

Forced Vital Capacity, Forced Expiratory Volume In 1s, and MIP will be measured following the recommendations proposed by the American Thoracic Society guidelines.

**Diaphragmatic function**

The diaphragmatic function will be reflected by the diaphragmatic thickness. The bilateral diaphragmatic thickness will be measured by ultrasound machines (Mindray M9, Shenzhen, China).

**Balance function**

The balance function of people after stroke will be reflected by Trunk Impairment Scale (TIS), Timed Up and Go Test (TUG), Falls Efficacy Scale–International (FES-I), and changes in center of pressure (COP) in sitting tests. The COP will be measured using a force plate (Sensor Medica, Guidonia Montecelio, Roma, Italy).

**Excise capacity**

The exercise capacity of patients will be measured by the 6-minute walk test (6MWT).

**Quality of life**

The Stroke Impact Scale 3.0 (SIS) of the Chinese version will be used to measure the quality of life.

**Data analysis**

All data will be analyzed using the IBM SPSS Statistics for Windows, Version 25.0 (Armonk, NY: IBM Corp).

Repeated-measures ANOVA will be performed to compare the changes of lung function, diaphragmatic function, balance function, exercise capacity and quality of life, respectively.

If the final data for any outcome do not meet the Gaussian assumption, non-parametric tests (e.g., Friedman's test) will be used to compare the differences.

A significance level of 0.05 will be applied for all analyses.
