# Supplementary material for: Effects of inspiratory muscle training on respiratory function, diaphragmatic thickness, balance control, exercise capacity and quality of life in people after stroke: A randomized controlled trial protocol
Source: PLoS One. 2025 Mar 25;20(3):e0319899. doi: 10.1371/journal.pone.0319899 (PMC11936291; doi:10.1371/journal.pone.0319899)
Supplement: S4 File — (DOCX) [file pone.0319899.s004.docx]

**S4 File. Adverse events or serious adverse events assessment and management**

Even though IMT has been shown to be safety employed in people after stroke, researchers are still obliged to take necessary measures to monitor safety and protect the participants. The following signs or symptoms will be monitored before, during, and after IMT intervention: dyspnea, headache, pain, tachycardia, hypertension, bronchospasm, dizziness, syncope, epistaxis (nose bleed), and respiratory failure. Any occurrence of above symptoms during the intervention exercises will be noted and recorded, including the start date, stop date, description, severity, and amount.

If adverse events / serious adverse events occur during the trial, appropriate treatment measures will be given to the patients immediately. The Ethics Committees of the hospital and university will be informed. Adverse events will be meticulously recorded in the case report form (CRF), with an assessment of their relation to the intervention. Any suspension of treatment will be noted with the reason in the CRF. Data on adverse events will be appropriately analyzed and incorporated into the final study report. During this process, whether patients' blinding status will be broken would be determined by the principal investigator (PI).
